# The CoQ biosynthetic di-iron carboxylate hydroxylase COQ7 is inhibited by in vivo metalation with manganese but remains functional by metalation with cobalt

**DOI:** 10.17912/micropub.biology.000635

**Published:** 2022-09-12

**Authors:** Ying Wang, Siegfried Hekimi

**Affiliations:** 1 Department of Biology, McGill University, Montreal, Quebec, Canada

## Abstract

Coenzyme Q (CoQ; ubiquinone) is an obligate component of the mitochondrial electron transport chain. COQ7 is a mitochondrial hydroxylase that is required for CoQ biosynthesis. COQ7 belongs to di-iron carboxylate enzymes, a rare type of enzyme that carries out a wide range of reactions. We found that manganese exposure of mouse cells leads to decreased COQ7 activity, but that pre-treatment with cobalt interferes with the inhibition by manganese. Our findings suggest that cobalt has greater affinity for the active site of COQ7 than both iron and manganese and that replacement of iron by cobalt at the active site preserves catalytic activity.

**Figure 1. Mn 2+ exposure results in a decrease of COQ7 activity, which can be inhibited by Co 2+ .**
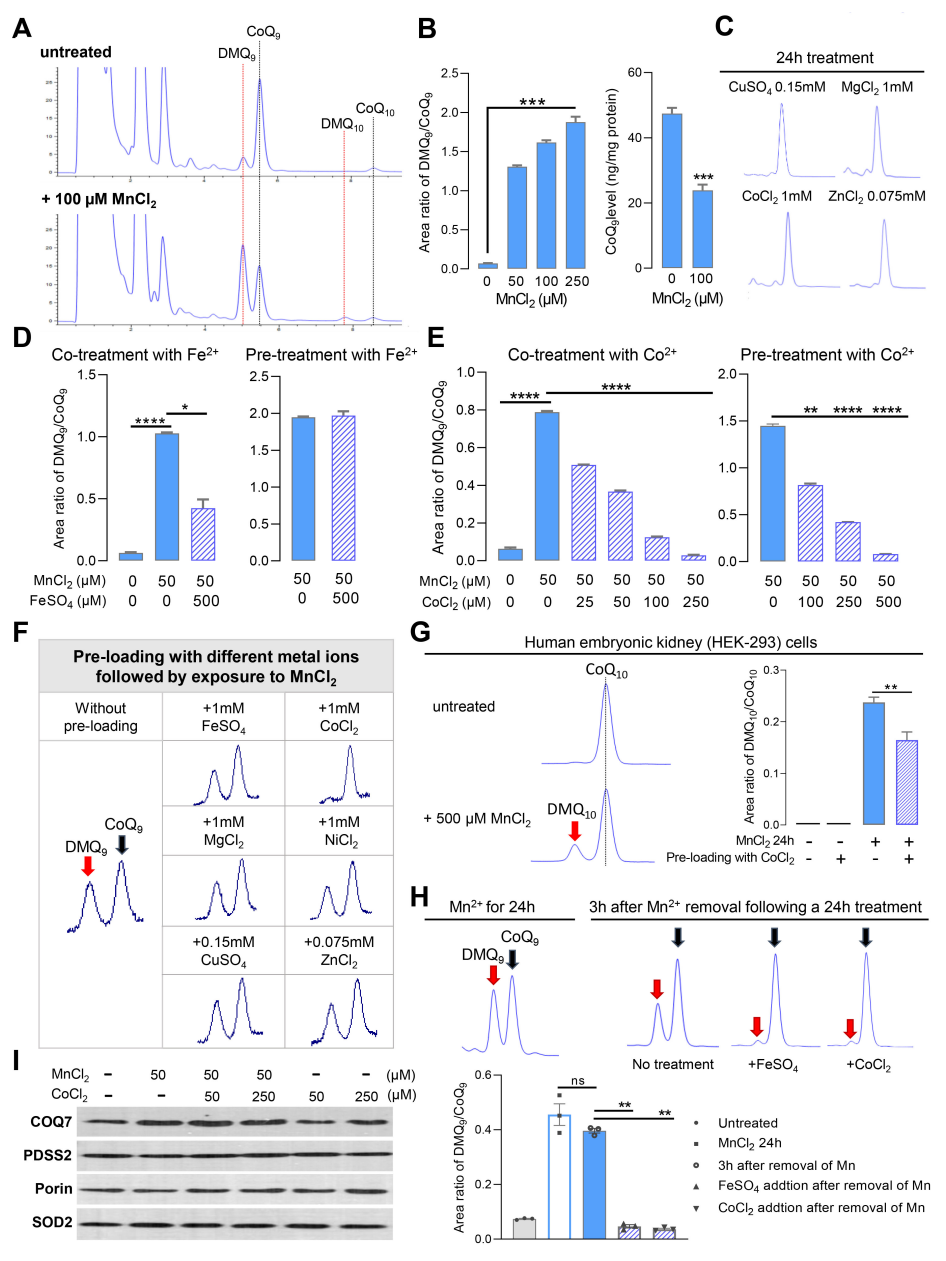
( **A** ) High-performance liquid chromatography (HPLC) traces of CoQ extracts from MnCl _2_ -treated or untreated RAW264.7 cells. ( **B** ) Increased DMQ _9_ :CoQ _9_ ratio and CoQ _9_ loss relative to protein levels after MnCl _2_ treatment. Data are shown as mean ± SEM of 2-3 replicates. * *p* <0.05; *** *p* <0.001 versus untreated control (one-way ANOVA followed by Dunnett's post hoc or Student’s *t* -test). MnCl _2_ was added to the culture medium for 24h before harvesting for CoQ extraction. ( **C** ) Representative HPLC traces of CoQ _9_ peaks in quinone extracts from RAW264.7 cells after 24h treatment with different metal ions. The maximum dose tolerated for 24h treatment was used for each tested metal. None of the indicated treatments resulted in the appearance of DMQ, indicating a lack of effect on COQ7 activity. ( **D** ) Effects of co-treatment or pre-loading with FeSO _4_ on the inhibition of COQ7 activity induced by MnCl _2_ exposure. **(E)** Effects of co-treatment or pre-loading with CoCl _2_ on the inhibition of COQ7 activity induced by MnCl _2_ exposure. Values are mean ± SEM (n=3) and the statistical analysis was performed by one-way ANOVA followed by Tukey's or Dunnett's post hoc test (* *p* <0.05; ** *p* <0.01; *** *p* <0.001; **** *p* < 0.0001). **(F)** Effects of pre-loading with different metals on the inhibition of COQ7 activity by Mn ^2+^ . RAW 264.7 cells were treated with the indicated metal ions for 3h before the metal ions were removed from the media and the cells were cultured for an additional 24h with MnCl _2_ (50 µM). Representative HPLC traces of DMQ _9_ and CoQ _9_ peaks are shown where a DMQ _9_ peak is followed by a CoQ _9_ peak, except that under Co ^2+^ pre-loading condition only the CoQ _9_ peak is visible. **(G)** DMQ _10_ appearance in human embryonic kidney (HEK-293) cells after exposure to Mn ^2+^ . HEK-293 cells were treated with 0.5mM MnCl _2_ for 24h before CoQ extraction. To test the effect of Co ^2+^ pre-loading, HEK-293 cells were treated with 1mM of CoCl _2_ for 3h followed by removal of Co ^2+^ from the medium, washing, and treatment with Mn ^2+^ for 24h. Error bar is SEM, n = 3. ** *p* < 0.01 (Student's *t* -test). **(H)** Supplementing FeSO _4_ or CoCl _2_ in the medium after removal of Mn ^2+^ results in accelerated recovery of COQ7 activity. RAW264.7 cells were collected either after 24h of Mn ^2+^ treatment, or 3h after Mn ^2+^ washout (following a 24h treatment), with or without addition of Fe ^2+^ or Co ^2+^ . MnCl _2_ : 50 µM. After removal of Mn ^2+^ , metals were added to the medium at a dosage of 500 µM. Error bar is SEM, n = 3. ** *p* < 0.01 (one-way ANOVA with Tukey's multiple comparison test). ns: not significant. ( **I** ) Western blot analysis of COQ7.

## Description

COQ7 is the penultimate enzyme in the coenzyme Q (CoQ) biosynthetic pathway. CoQ, also known as ubiquinone, is an endogenously synthesized, highly hydrophobic, lipid-soluble molecule found in virtually all animal cells as well as in plants and microbes (Wang and Hekimi 2013, Liu and Lu 2016). It is a pivotal component of the mitochondrial respiratory chain, where it functions as an obligate electron carrier, enabling ATP generation from oxidative phosphorylation (Turunen, Olsson et al. 2004). Loss of sufficient CoQ biosynthesis is associated with diverse severe pathologies (Quinzii and Hirano 2011, Wang and Hekimi 2013, Doimo, Desbats et al. 2014, Hughes, Harrison et al. 2017). The CoQ biosynthetic pathway is highly conserved from yeast to humans. The final steps are carried out in the mitochondria (Stefely and Pagliarini 2017, Hajj Chehade, Pelosi et al. 2019, Wang and Hekimi 2019). A deficiency of COQ7 in animal cells results in accumulation of its substrate, demethoxyubiquinone (DMQ) (Levavasseur, Miyadera et al. 2001, Nakai, Yuasa et al. 2001, Garcia-Corzo, Luna-Sanchez et al. 2013, Wang and Hekimi 2013, Wang, Oxer et al. 2015, Wang, Smith et al. 2017, Hidalgo-Gutierrez, Barriocanal-Casado et al. 2019, Wang, Gumus et al. 2022). Structurally, COQ7 has been identified to be a carboxylate-bridged di-iron enzyme (Stenmark, Grunler et al. 2001, Berthold and Stenmark 2003, Behan and Lippard 2010). Consistent with this, we previously reported that exposure to the metal-chelating agent clioquinol inhibits CLK-1/COQ7 activity due to iron chelation (Wang, Branicky et al. 2009).

In this present study, we report that exposure of RAW264.7 cells to Mn ^2+^ ions results in CoQ loss and accumulation of DMQ, which indicates COQ7 inhibition ( **Fig. 1A, B** ). CoQ is composed of a benzoquinone ring and a lipophilic poly-isoprenoid tail whose chain length varies according to species. Mouse cells have mostly CoQ _9_ (9 referring to the number of isoprene units in the tail) but also a small amount of CoQ _10_ . We observed similar effects of Mn ^2+^ exposure on CoQ _9_ and CoQ _10_ ( **Fig. 1A** ). Of note, as we extracted DMQ _9_ and CoQ _9_ from the same cells, we simply used the DMQ _9_ to CoQ _9_ ratio as the best measure of COQ7 enzymatic activity. As shown in **Fig. 1B** , Mn ^2+^ treatment results in a dose-dependent increase of the DMQ _9_ /CoQ _9_ ratio and 2-fold lower CoQ _9_ levels relative to protein content, while no significant effect on cell viability was observed. In contrast, no other metals tested, including Cu ^2+^ , Mg ^2+^ , Co ^2+^ , and Zn ^2+^ were found to cause accumulation of DMQ ( **Fig. 1C** ).

Our finding suggests that Mn toxicity acting on COQ7 could lead to CoQ deficiency in vivo. Note however that our experiments cannot conclude that Mn ^2+^ does not also affect the total level of CoQ by some other mechanism as well. The overall consequences on cell and organism function of CoQ deficiency induced by excess Mn ^2+^ exposure however remain to be determined in future studies. Divalent ions of Mn and of Fe are similar in chemistry, and both tend to coordinate with six ligands and form octahedral complexes (Cotruvo and Stubbe 2012). This makes it difficult for proteins to discriminate between Fe ^2+^ and Mn ^2+^ on the basis of structure alone (Cotruvo and Stubbe 2012). Therefore, we speculate that inhibition of COQ7 activity by Mn ^2+^ was due either to interference with Fe ^2+^ uptake and/or by binding to COQ7 instead of Fe ^2+^ . To test this, we first determined the effect on COQ7 activity of simultaneous exposure to Mn ^2+^ and Fe ^2+^ . We found that Fe ^2+^ co-treatment significantly prevents the inhibitory effect of Mn ^2+^ on COQ7 activity ( **Fig. 1D** ). To explore this further, we pretreated the cells for 3h with Fe ^2+^ to increase the intracellular availability of Fe; then, after removal of Fe ^2+^ from the media, the cells were treated with Mn ^2+^ for a total of 24h before CoQ extraction. Under these conditions, the cells should have plenty of Fe ^2+^ , yet this had no effect on the inhibitory effect of Mn ^2+^ on COQ7 ( **Fig. 1D** ). This result suggests the possibility of a preference for Mn ^2+^ over Fe ^2+^ for binding to the metal binding site of the enzyme.

In addition, we found that co-treatment with Co ^2+^ also prevents Mn ^2+^ from inhibiting COQ7, perhaps even more efficiently than Fe ^2+^ ( **Fig. 1E** ). As with Fe ^2+^ , this could be due to competition with Mn ^2+^ for uptake. However, surprisingly, and in contrast to what we saw with Fe ^2+^ , pre-loading cells with Co ^2+^ prevented the inhibitory effect of Mn ^2+^ on COQ7 ( **Fig. 1E** ). All the other metals tested, however, including Cu ^2+^ , Mg ^2+^ , Ni ^2+^ and Zn ^2+^ were, like Fe ^2+^ , unable to block the effect of Mn ^2+^ ( **Fig. 1F)** . Co ^2+^ has similar chemical properties as those metals in the same group on the periodic table, including Mn ^2+^ and Fe ^2+^ . However, it occurs much less frequently in metalloproteins, likely due to its low abundance in nature (Okamoto and Eltis 2011). Mis-metalation with Co ^2+^ at protein binding sites has been shown previously (Okamoto and Eltis 2011). We postulate that the way Co ^2+^ prevents Mn ^2+^ from inhibiting COQ7 is that Co ^2+^ can physically and functionally replace the lower order metals (Fe ^2+^ and Mn ^2+^ ) at the catalytic site of COQ7, at least under the condition of an excess of Co ^2+^ . More specifically, as Co ^2+^ lies towards the tighter binding end of the Irving–Williams series compared to Mn ^2+^ , the binding of Co ^2+^ to the di-iron cluster of COQ7 would render subsequent Mn ^2+^ treatment less effective at inhibiting COQ7, meaning that Mn ^2+^ is not be able to compete out Co ^2+^ due to its relatively lower affinity for binding. Treatment of human embryonic kidney cells (HEK-293) with Mn ^2+^ also led to reduced activity of COQ7, and Co ^2+^ pre-loading prevented the effect ( **Fig. 1G)** .

Furthermore, we observed that, while COQ7 activity only slowly recovers after the removal of Mn ^2+^ from the medium, addition of Fe ^2+^ in the medium immediately after Mn ^2+^ washout significantly accelerates the recovery of COQ7 activity ( **Fig. 1H** ). This is consistent with our hypothesis that inhibition of COQ7 activity by Mn ^2+^ is by substituting for Fe at the di-iron active site of the enzyme. Co ^2+^ addition shows the same effect ( **Fig. 1H** ), which is again consistent with the notion that Co ^2+^ is as functional as Fe ^2+^ at the active site of COQ7. In this model, after interruption of Mn ^2+^ treatment, the presence of Co ^2+^ would allow Mn replacement by Co, leading to rapid restoration of COQ7 activity. As expected, the other tested metals, including Cu ^2+^ , Mg ^2+^ , and Zn ^2+^ showed no effect on the course of the recovery of the COQ7 activity after Mn ^2+^ exposure was terminated.

Lastly, we used Western blot analysis to determine whether the inhibition of COQ7 activity by Mn ^2+^ is associated with a change of COQ7 expression level. The COQ7 level was found to be higher in Mn ^2+^ -treated cells and the change could not be prevented by co-treatment with Co ^2+^ , despite its ability to maintain COQ7 functionality ( **Fig. 1I** ). The effect of Mn ^2+^ is specific for COQ7 as the level of PDSS2 was not affected ( **Fig. 1I** ). PDSS2 is another COQ-biosynthetic enzyme, which catalyzes the assembly of the polyisoprenoid side chain. Co ^2+^ treatment at a relatively high dose (250 µM), also leads to elevation of COQ7 concentration. We speculate that the increase of COQ7 level after Mn ^2+^ or Co ^2+^ exposure could be due to increased COQ7 protein stability because of Mn or Co at the enzyme’s active center.

Metal ions play key structural and functional roles for nearly half of all known proteins. A better understanding of how metalation is controlled and how it modulates the functions of proteins is important. COQ7 presents a unique opportunity to look at in vivo activity with different metals because of the ease with which the reaction products can be monitored in vivo. The sensitivity of cellular COQ7 to metals appears to be greater in the RAW264.7 macrophage line than in HEK-293 cells, possibly because of a greater propensity of macrophages to take up and accumulate metals. Nevertheless, we believe that the present study is the first to describe metal swapping in a di-iron carboxylate enzyme, which are enzymes that are of commercial and ecological interest (Sirajuddin and Rosenzweig 2015, Blanchette, Knipe et al. 2016, Tveit, Hestnes et al. 2019). It warrants further studies and research into the metalation and mismetalation of di-iron carboxylate proteins.

## Methods

Cell culture

RAW264.7 were cultured in high glucose DMEM (Dulbecco's modified Eagle's medium) supplemented with 10% fetal bovine serum and 1% antibiotic/antimycotic mix at 37°C in a humidified atmosphere of 95% air and 5% CO _2_ . For single reagent treatments or co-treatment experiments, reagents were added once to the culture in 6 well plates and cells were harvested after 24h of incubation. In pre-treatment experiments, cells were exposed to different metal ions for 3h, washed with phosphate buffered saline (PBS) and then treated with MnCl _2_ for 24 hours. To test the effects of different metal ions on recovery of COQ7 activity after MnCl _2_ removal, MnCl _2_ - supplemented medium was removed after 24h of incubation. Following washes in PBS, fresh media containing different metal salts were added and CoQ was extracted at 3h after the Mn ^2+^ washout. All chemicals were obtained from Sigma-Aldrich.

Extraction and high-performance liquid chromatography (HPLC) determination of CoQ

CoQ extraction and quantitation using HPLC were performed as described previously. Briefly, cell lysates were prepared in a radioimmunoprecipitation buffer (20mM Tris-HCl, pH 7.5, 1% NP-40, 0.5% deoxycholate, 10 mM EDTA, 150 mM NaCl) and CoQ were extracted with ethanol and hexane (v/v 2/5). An Agilent 1260 Infinity LC system equipped with a quaternary pump (G7111A) and a variable wavelength detector (G7114A) was used. Chromatography was carried out on a reverse-phase C18 column (2.1 x 50 mm, 1.8 µm, Agilent) with 70% methanol and 30% ethanol as the mobile phase at a flow rate of 0.3 mL/min. The detector was set at 275 nm. The CoQ _9_ peak was identified using pure CoQ _9_ . The DMQ _9_ peak was identified by comparing to quinone extraction from *Coq7* knockout mouse embryonic fibroblasts (MEFs) (Levavasseur, Miyadera et al. 2001, Wang and Hekimi 2013).

Western blot

Protein samples were prepared in RIPA buffer, and 50 µg of lysates were subjected to 12% SDS-PAGE and visualized by using antibodies against COQ7, PDSS2, SOD2, or Porin/VDAC. Detection was performed with ECL reagent and exposed to x-ray film.

Statistical analysis

Statistical analysis and graphical presentation were carried out using GraphPad Prism 9.2 software. A *p* -value of < 0.05 was considered significant for all tests.

## Reagents

**Table:** 

**NAME**	**AVAILABLE FROM**
RAW264.7 cell line	ATCC
Dulbecco's modified Eagle's medium	Wisent, Inc
Fetal bovine serum	Wisent, Inc
antibiotic/antimycotic mix	Wisent, Inc
RIPA buffer	Cell Signaling Technology
Anti-COQ7 antibody	ProteinTech (15083-1-AP)
Anti-PDSS2 antibody	ProteinTech (13544-1-AP)
Anti-SOD2 antibody	Abcam (ab227091)
Anti-Porin/VDAC antibody	Cell Signaling Technology ((#4661)
ECL substrates	FroggaBio (NEL103001EA)
